# Clinical Significance and Inflammatory Landscape of aNovel Recurrence-Associated Immune Signature in Stage II/III Colorectal Cancer

**DOI:** 10.3389/fimmu.2021.702594

**Published:** 2021-07-29

**Authors:** Zaoqu Liu, Taoyuan Lu, Jing Li, Libo Wang, Kaihao Xu, Qin Dang, Long Liu, Chunguang Guo, Dechao Jiao, Zhenqiang Sun, Xinwei Han

**Affiliations:** ^1^Department of Interventional Radiology, The First Affiliated Hospital of Zhengzhou University, Zhengzhou, China; ^2^Interventional Institute of Zhengzhou University, Zhengzhou, China; ^3^Interventional Treatment and Clinical Research Center of Henan Province, Zhengzhou, China; ^4^Department of Cerebrovascular Disease, Zhengzhou University People’s Hospital, Zhengzhou, China; ^5^Department of Hepatobiliary and Pancreatic Surgery, The First Affiliated Hospital of Zhengzhou University, Zhengzhou, China; ^6^Department of Colorectal Surgery, The First Affiliated Hospital of Zhengzhou University, Zhengzhou, China; ^7^Department of Endovascular Surgery, The First Affiliated Hospital of Zhengzhou University, Zhengzhou, China

**Keywords:** stage II/III colorectal cancer, recurrence, immune signature, immunotherapy, adjuvant chemotherapy, immune checkpoints

## Abstract

**Background:**

A considerable number of patients with stage II/III colorectal cancer (CRC) will relapse within 5 years after surgery, which is a leading cause of death in early-stage CRC. The current TNM stage system is limited due to the heterogeneous clinical outcomes displayed in patients of same stage. Therefore, searching for a novel tool to identify patients at high recurrence-risk for improving post-operative individual management is an urgent need.

**Methods:**

Using four independent public cohorts and qRT-PCR data from 66 tissues, we developed and validated a recurrence-associated immune signature (RAIS) based on global immune genes. The clinical and molecular features, tumor immune microenvironment landscape, and immune checkpoints profiles of RAIS were also investigated.

**Results:**

In five independent cohorts, this novel scoring system was proven to be an independent recurrent factor and displayed excellent discrimination and calibration in predicting the recurrence-risk at 1~5 years. Further analysis revealed that the high-risk group displayed high mutation rate of TP53, while the low-risk group had more abundance of activated CD4+/CD8+ T cells and high expression of PD-1/PD-L1.

**Conclusions:**

The RAIS model is highly predictive of recurrence in patients with stage II/III CRC, which might serve as a powerful tool to further optimize decision-making in adjuvant chemotherapy and immunotherapy, as well as tailor surveillance protocol for individual patients.

## Introduction

By 2020, colorectal cancer (CRC) has become the third most prevalent cancer and second leading cause of cancer-related mortality globally (1). The American Joint Committee on Cancer (AJCC) and Union for International Cancer Control (UICC) TNM stage system is usually applied to manage the prognosis and adjuvant chemotherapy of early-stage CRC (2). 5-Fluorouracil (5-FU)-based adjuvant chemotherapy aims to eliminate residual cancer cells after surgical resection, thus reducing the recurrence rate or extending the time to recurrence. It is a conventional therapy for stage III and a subset of stage II CRC patients (e.g., T4, high grade) (3, 4). However, administration of adjuvant chemotherapy is unsatisfactory in clinical practice, with many patients not benefiting from this because they are either cured by surgery or relapse after adjuvant chemotherapy (5). Moreover, current guidelines demonstrate that the present definition of “high-risk” stage II CRC remains insufficient (6). Previous studies have shown that approximately half of stage III CRC patients will relapse within 5 years after surgical resection, while the 5-years recurrence rate of stage II CRC patients is about 12~38% (7–9). In clinical practice, the AJCC stage system alone is limited in stratifying these patients (10), thus, searching for new ways to identify patients at high risk for recurrence who have urgent need for additional therapeutic schemes is imperative.

Indeed, substantial efforts have been put into identifying and developing markers for assessing the recurrence-risk in early-stage CRC. The consensus molecular subtype (CMS) system has been reported to correlate with clinical outcomes in stage II/III CRC, CMS4 tumors have dismal recurrence and overall survival (11). In parallel, the transcriptomic-based CRC intrinsic subtype (CRIS) that reveals cancer-cell intrinsic characteristics has been proven to be an independent factor of prognosis, and CRIS-C tumors are superior at risk of recurrence (12). Patients with high level of microsatellite instability (MSI-H) are more prone to show a dramatically reduced risk of death and recurrence (13). Recently, circulating tumor DNA analyses have been demonstrated to serve as biomarkers of recurrence and benefit of adjuvant therapy in stage III CRC (14). In addition, alterations in various genes, such as *BRAF*, *KRAS*, and *PIK3CA* mutations, *SMAD4* deletion, and *HER2* amplification, are also significantly associated the recurrence after curative surgery of early-stage CRC (15–19). We have previously reported that *TTN*/*OBSCN* “Double-hit” suggests favorable prognosis in CRC (20). Nonetheless, these markers only have a moderate prediction accuracy and limited clinical usefulness (21, 22).

In recent years, tumor immune microenvironment (TIME) markers have displayed encouraging progress in evaluating prognostic and immunotherapy of patients (22–24). In-situ adaptive immune cells infiltration (e.g., CD8+ cytotoxic T lymphocyte) is linked with the time to recurrence (25). Actually, an immunohistochemistry-based scoring pipeline has been established and validated (termed Immunoscore^®^), which quantifies the densities of two adaptive immune cells, CD3+ and CD8+ T cells, in the core and invasive margin of tumor (22). Although Immunoscore^®^ displays a stable predictive power of prognosis in early-stage CRC, and has been introduced as a diagnostic standard in the 5^th^ edition of the WHO Digestive System Tumors (26), its performance remains at a moderate accuracy of Harrell’s C-statistics ranging from 0.56 to 0.68 in international researches (22). This may be due to the fact that only two adaptive immune cells are considered, but other immune components have been also reported to correlate with the time to death and recurrence, such as macrophages, nature killer (NK) cells, and γδ T lymphocytes (27–29). Therefore, estimating the recurrence-risk of patients with stage II/III CRC based on the global immune milieu might improve the accuracy of model. Traditional techniques such as immunohistochemistry or quantitative real-time polymerase chain reaction (qRT-PCR) are difficult to quantify a broad spectrum of immune genes, but advances in bioinformatics and machine learning have made it possible. With the help of machine learning, such as the least absolute shrinkage and selection operator (LASSO) algorithm, it is easy to identify the most important elements based on the expression profiles of global immune genes and fit a model with strong generalization performance (30).

In this study, using four independent public cohorts with gene expression and recurrence-free survival (RFS) data, we developed and validated an individualized recurrence-associated immune signature (RAIS) for stage II/III CRC after surgical resection. The clinical and molecular features, TIME landscape, and immune checkpoints profiles of RAIS were also investigated. Furthermore, we used 66 frozen tissue samples with qRT-PCR data for experimental verification to prove the stability and reliability of the RAIS model. Initial construction of the RAIS for stratifying the recurrence-risk will enhance the understanding of underlying mechanisms between immune molecules and the recurrence of CRC, and might help optimize decision-making in adjuvant chemotherapy and immunotherapy for patients with stage II/III CRC.

## Materials and Methods

### Publicly Available mRNA Data and Immune Gene Sets

We retrospectively collected four CRC cohorts from The Cancer Genome Atlas (TCGA, https://portal.gdc.cancer.gov) and Gene Expression Omnibus (GEO, http://www.ncbi.nlm.nih.gov/geo) databases, including TCGA-CRC, GSE143985, GSE29621, and GSE92921. The data processing was described in detail in the Supplementary Method. In four cohorts, we only retained CRC patients that met the following criteria: (1) Have mRNA expression data; (2) Have both recurrent status and RFS information; (3) In the AJCC stage II/III; (4) No preoperative chemotherapy or radiotherapy received. A total of 171 patients from TCGA-CRC were used as the training set, and GSE143985 (n =91), GSE29621 (n =40), and GSE92921 (n =59) from the GEO database were used as the validation sets. The corresponding clinical information of four cohorts was also downloaded, and the baseline data were summarized in **Table S1**. The list of immune-related genes (IRGs) was retrieved from ImmPort (https://www.immport.org), IRIS (http://www.immunegene.org), and InnateDB (https://www.innatedb.com) databases. There were a total of 2504 IRGs that could be detected in all four cohorts.

### Signature Generation

First, based on univariate Cox regression, we identified stable recurrence-associated IRGs using the following criteria: (1) *P <*0.05 in at least half of the enrolled cohorts; (2) all hazard ratio (HR) greater or less than 1 in the cohorts with statistical significance. Second, using the expression of these stable recurrence-associated IRGs in TCGA-CRC, we fitted a LASSO Cox regression model for assessing the RFS of patients with stage II/III CRC. Using the 10-fold cross validations, the optimal lambda was obtained when the partial likelihood deviance reached the minimum value. Third, based on the optimal lambda, the IRGs with nonzero coefficients were selected to construct the prediction signature. The risk score for each patient was calculated with the LASSO model weighting coefficient as follows:

$$Risk\;score=\sum_{i=1}^{n}Exp_{i}\times Coef_{i}$$

where *n* is the number of key IRGs, *Exp_i_* is the expression of IRG *i*, and *Coef_i_* is the LASSO coefficient of IRG *i*.

### TIME Characterization Analysis

Gene expression profiles were utilized to decipher the TIME characterization of CRC samples with multiple bioinformatics tools. The immunophenoscore (IPS) was applied to assess the immune state of each sample. IPS is a scoring scheme that quantifies the immunogenicity of tumor samples using a variety of markers of immune response or immune tolerance. The higher the IPS z-score, the stronger the immunogenicity of the sample (31). To measure the infiltration abundance of immune cell populations in tumor tissues, two different tools were applied. First, we leveraged the R package ESTIMATE to infer the fraction of stromal and immune cells in CRC samples, and generated two scores including the immune and stromal scores (32). Next, to describe a more detailed landscape of immune cell types infiltration, we employed the single sample gene set enrichment analysis (ssGSEA) implemented in R package GSVA. The gene sets for marking each cell were obtained from the research of Charoentong, which stored 28 human immune cell subtypes (31).

In addition, to predict their putative response to immune checkpoint blockade (ICB), CRC samples were scored using T-cell inflammatory signature (TIS) and Tumor Immune Dysfunction and Exclusion (TIDE) approaches. TIS proposed by Ayers et al. was used to predict clinical response to *PD-1* blockade. The signature was composed of 18 inflammatory genes associated with antigen presentation, chemokine expression, cytotoxic activity, and adaptive immune resistance (33). The TIDE algorithm (http://tide.dfci.harvard.edu/) integrates the expression signature of two primary mechanisms of immune evasions: T cell dysfunction and T cell exclusion, to model tumor immune evasion. Patients with higher TIDE score suggest the greater potential of tumor immune evasion; thus, these patients would derive worse immunotherapy response (34).

### Patients and Tissue Specimens

From January 2015 to December 2016, we collected a total of 66 frozen surgically resected CRC tissues with AJCC stage II/III at The First Affiliated Hospital of Zhengzhou University. Follow up was concluded five years after surgery, and all patients gave written informed consent. Detailed baseline data of CRC patients were displayed in **Table S1**. Total RNA was isolated from CRC tissues using RNAiso Plus reagent (Takara, Dalian, China) according to the manufacturer’s instructions. RNA quality was evaluated using a NanoDrop One C (Waltham, MA, USA), and RNA integrity was assessed using agarose gel electrophoresis. An aliquot of 1 μg of total RNA was reverse transcribed into complementary DNA (cDNA) according to the manufacturer’s protocol using a High-Capacity cDNA Reverse Transcription kit (TaKaRa BIO, Japan). All cDNA samples were prepared for qRT-PCR. This project was approved by the Ethics Committee Board of The First Affiliated Hospital of Zhengzhou University.

### qRT-PCR Analysis

In the qRT-PCR analysis, the enrolled 12 genes in the RAIS signature and feature genes (including *PD-L1*, *PD-1*, *CD4* and *CD8A*) were detected. qRT-PCR was performed using SYBR Assay I Low ROX (Eurogentec, USA) and SYBR^®^ Green PCR Master Mix (Yeason, Shanghai, China). The expression value of the target genes was normalized to *GAPDH*, and then log2 transformed for subsequent analysis. The primer sequences of the included 12 genes and *GAPDH* were shown in **Table S2**.

### Statistical Analysis

All data processing, statistical analysis, and plotting were conducted in R 4.0.2 software. The time from surgery to cancer recurrence was defined as RFS. Correlations between two continuous variables were assessed *via* Spearman’s correlation coefficients. Fisher’s exact test or Pearson’s chi-squared test was applied to compare categorical variables. Continuous variables were compared between two groups through the Wilcoxon rank-sum test or T test. The patients were divided into high and low-risk groups based on the median risk score. The Kaplan-Meier method and the log-rank test were utilized to estimate the different RFS between two groups. The receiver operating characteristic curve (ROC) used to predict binary categorical variables was implemented using the R package pROC. The time-dependent area under the ROC (AUC) for survival variable were conducted by the R package timeROC. The R package rms was applied to plot calibration curves. GO and KEGG enrichment analysis were performed by the R package clusterProfiler. All statistical tests were two-sided. *P <*0.05 was regarded as statistically significant.

## Results

### Patients’ Demographic Characteristics

As illustrated in **Table S1**, we collected a total of 427 patients with stage II/III CRC from five independent multicenter cohorts. In TCGA-CRC, there were 101 stage II and 70 stage III patients with a median RFS of 2.2521 years; the 1-, 2-, and 3-year recurrence rate were 5.3%, 8.2%, and 10.5%, respectively. In GSE143985, there were 55 stage II and 36 stage III patients with a median RFS of 5.8904 years; the 1-, 2-, and 3-year recurrence rate were 9.9%, 12.1%, and 15.4%, respectively. In GSE29621, there were 22 stage II and 18 stage III patients with a median RFS of 3.9379 years; the 1-, 2-, and 3-year recurrence rate were 7.5%, 10.0%, and 15.0%, respectively. In GSE92921, there were 43 stage II and 16 stage III patients with a median RFS of 5.7260 years; the 1-, 2-, and 3-year recurrence rate were 6.8%, 8.5%, and 10.2%, respectively. In qRT-PCR validation cohort, there were 40 stage II and 26 stage III patients with a median RFS of 3.9671 years; the 1-, 2-, and 3-year recurrence rate were 15.2%, 16.7%, and 21.2%, respectively.

### Construction and Validation of the RAIS With Stage II/III CRC in Public Datasets

**Figure 1:**
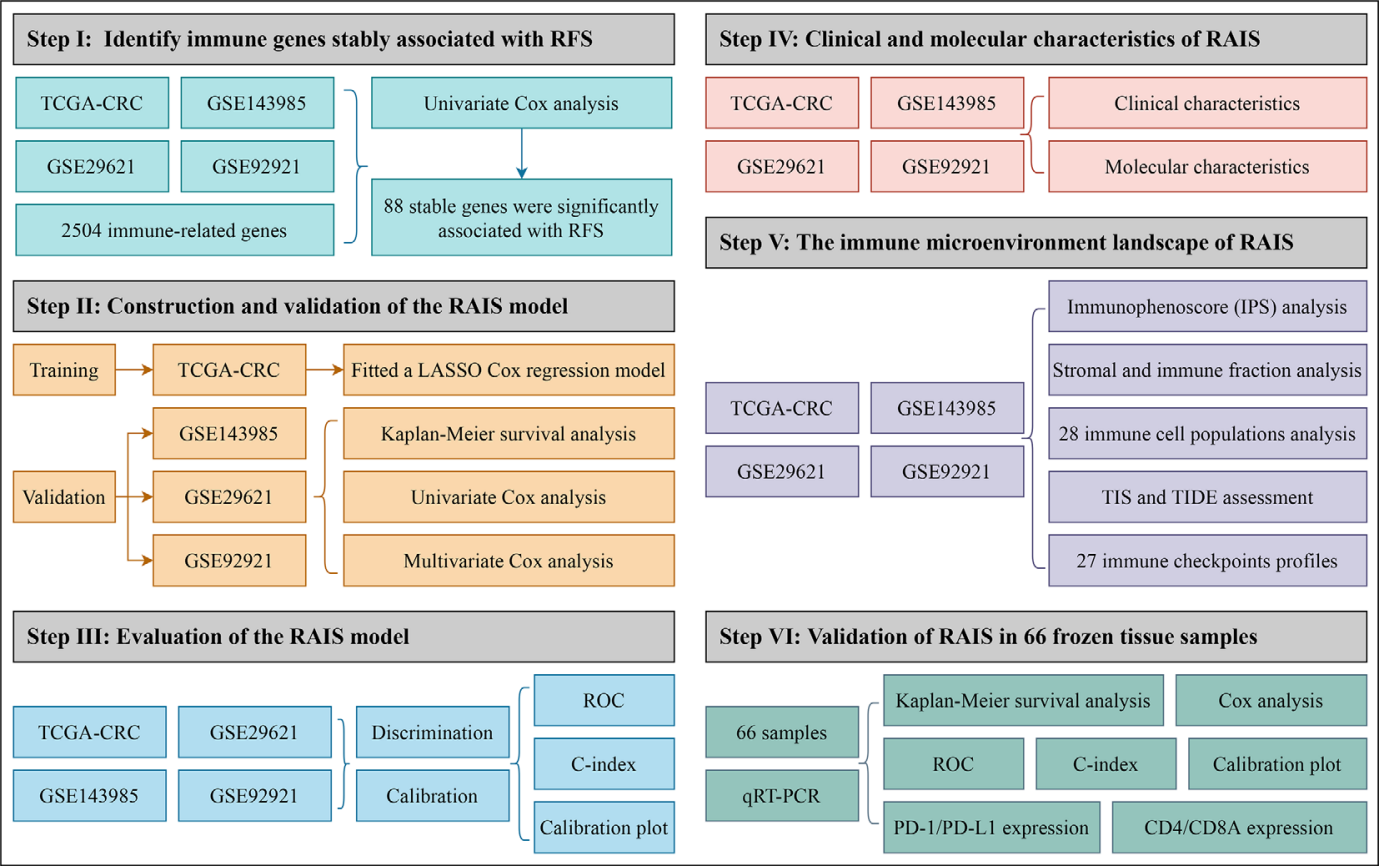
The flowchart of this study.

**Figure 2:**
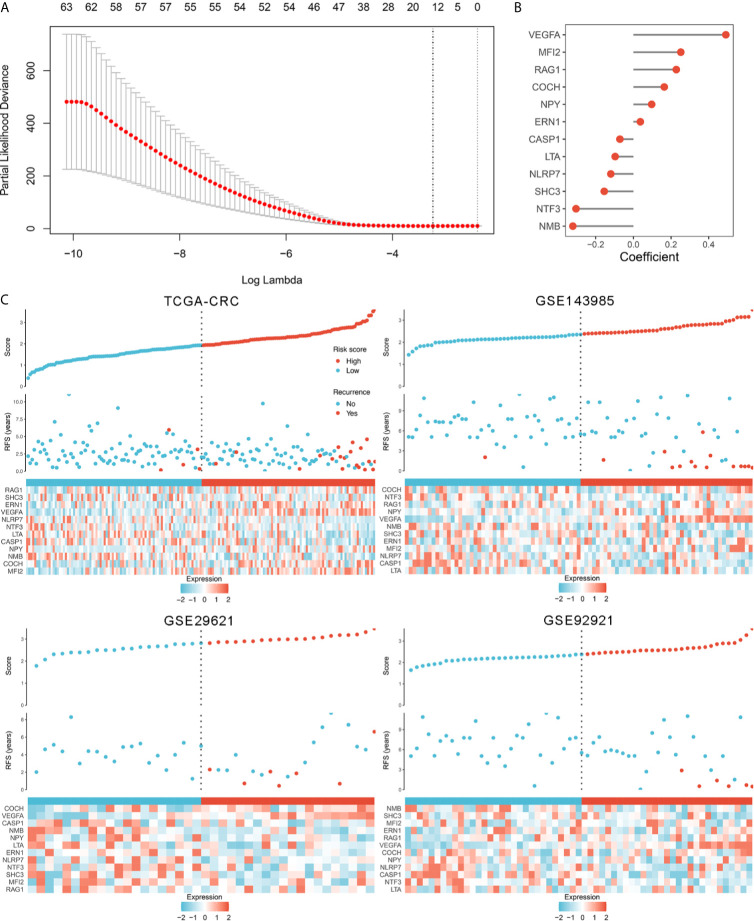
The development of the RAIS model based on the LASSO algorithm. **(A)** Ten-fold cross-validations to tune the parameter selection in the LASSO model. The two dotted vertical lines are drawn at the optimal values by minimum criteria (left) and 1−SE (standard error) criteria (right). **(B)** LASSO coefficient profiles of the candidate genes for RAIS construction. **(C)** The distribution of risk score, recurrence status, and gene expression panel in four cohort.

**Figure 3:**
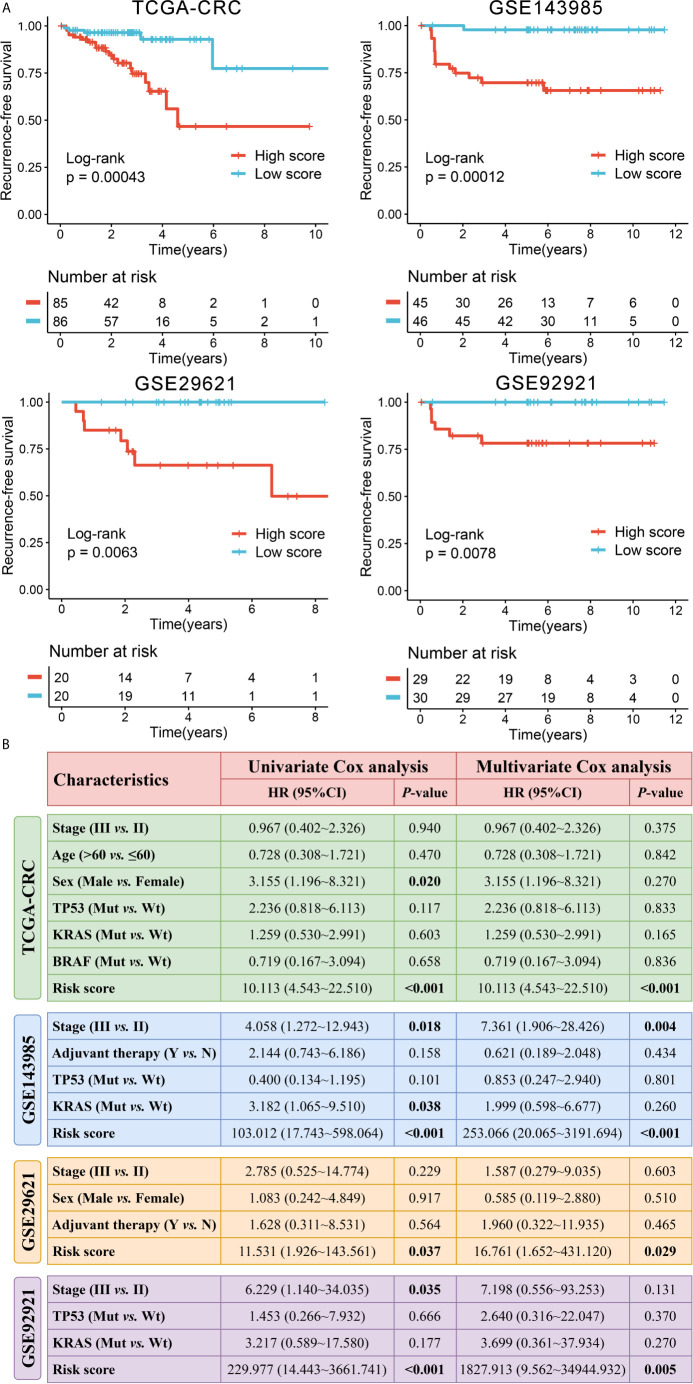
Survival significance of RAIS in four cohorts. **(A)** Kaplan-Meier curves of RFS according to the RAIS. **(B)** Univariate and multivariate Cox regression analysis of the risk score. The bold values mean P <0.05.

In order to comprehensively evaluate the relationships between IRGs and recurrence in patients with stage II/III CRC, an analysis pipeline was developed to display the process (**Figure 1**). Univariate Cox results of four cohorts revealed a total of 88 stable IRGs were significantly associated with RFS (**Table S3**). GO and KEGG enrichment analysis indicated these genes were mainly involved in immune response and inflammatory signaling pathways (**Figure S1**). Based on the expression of these genes in TCGA-CRC, we fitted a LASSO Cox regression model and identified 12 IRGs that were strongly predictive of RFS, including *MFI2*, *LTA*, *VEGFA*, *NPY*, *SHC3*, *RAG1*, *CASP1*, *NTF3*, *COCH*, *NMB*, *ERN1*, and *NLRP7* (**Figure 2A**). Next, a risk score for RAIS was calculated using a formula that including the 12 IRGs weighted by their regression coefficients in a penalized Cox model as follows: Risk score = 0.4906 × Exp(*VEGFA*) + 0.2516 × Exp(*MFI2*) + 0.2276 × Exp(*RAG1*) + 0.1638 × Exp(*COCH*) + 0.0970 × Exp(*NPY*) + 0.0369 × Exp(*ERN1*) - 0.0715 × Exp(*CASP1*) - 0.0967 × Exp(*LTA*) - 0.1198 × Exp(*NLRP7*) - 0.1547 × Exp(*SHC3*) - 0.3043 × Exp(*NTF3*) - 0.3210 × Exp(*NMB*) (**Figure 2B**). This formula was utilized to calculate the risk score for each patient in four cohorts. The expression heatmap of the 12 selected IRGs and the distribution of risk scores were illustrated in **Figure 2C**. All patients were assigned into high- and low-risk groups according to the median risk score (**Figure 2C**). Relative to the low-risk group, patients in the high-risk group had significantly dismal RFS in TCGA-CRC (HR =4.051, 95% confidence interval (95%CI) =2.497~6.571, log-rank *P* =0.00043), GSE143985 (HR =5.591, 95%CI =2.910~10.743, log-rank *P* =0.00012), GSE29621 (HR =2.317, 95%CI =1.536~4.854, log-rank *P* =0.0063), and GSE92921 (HR =6.823, 95%CI =2.568~18.133, log-rank *P* =0.0078) (**Figures 3A, B**). After controlling the available clinical characteristics in four cohorts, multivariate Cox regression analysis revealed RAIS remained an independent risk factor for evaluating RFS of stage II/III CRC patients (all *P <*0.05) (**Figure 3B**).

### Evaluation of the RAIS Model

**Figure 4:**
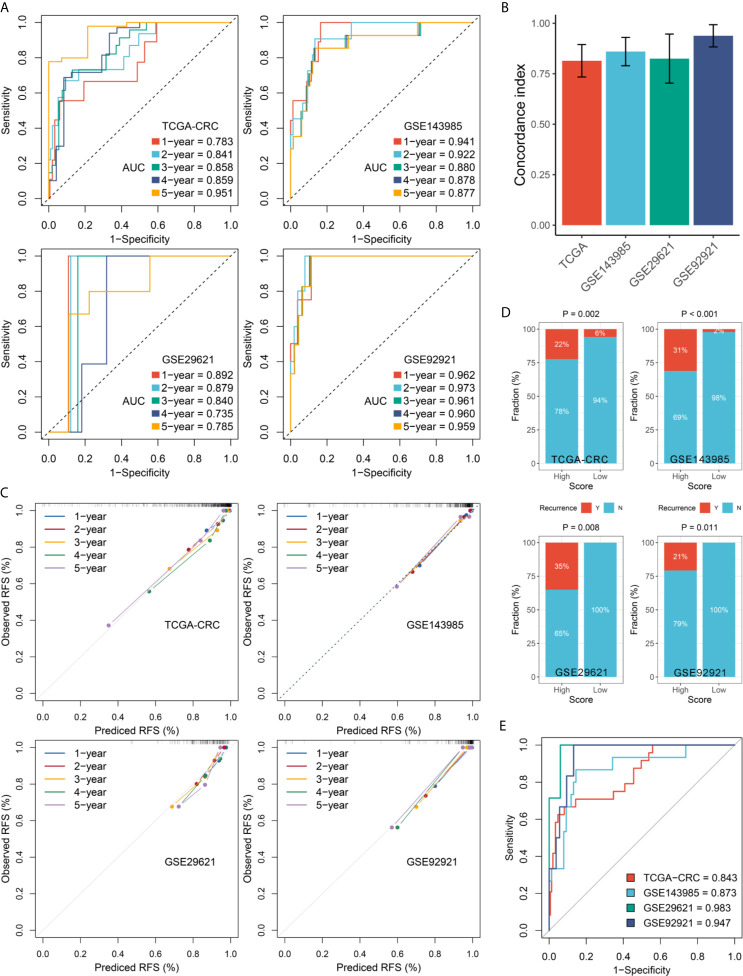
Evaluation of the RAIS model in four cohorts. **(A)** Time-dependent ROC analysis for predicting RFS at 1~5 years. **(B)** The Harrell’s C-index of RAIS. **(C)** Calibration plots for comparing the actual probabilities and the predicted probabilities of RFS at 1~5 years. **(D)** Comparison of recurrence rate between the high-risk and low-risk groups. **(E)** ROC analysis of the RAIS model for predicting the recurrence event of patients.

In this study, we evaluated this model from two perspectives: discrimination and calibration. The discrimination was assessed by ROC and Harrell’s C-index, and the calibration was assessed by calibration plots. The results showed that the AUCs for predicting RFS at 1~5 years was 0.783, 0.841, 0.858, 0.859, and 0.951 in TCGA-CRC, 0.941, 0.922, 0.880, 0.878, and 0.877 in GSE143985, 0.892, 0.879, 0.840, 0.735, and 0.785 in GSE29621, and 0.962, 0.973, 0.961, 0.960, and 0.959 in GSE92921, respectively (**Figure 4A**). The C-index were 0.814 (95%CI: 0.734~0.895), 0.860 (95%CI: 0.790~0.930), 0.825 (95%CI: 0.703~0.947), and 0.938 (95%CI: 0.883~0.993) in four cohorts, respectively (**Figure 4B**). The above indicated the high predictive accuracy of this model. Furthermore, the RAIS displayed excellent calibration, with the predicted probabilities of RFS at 1~5 years accurately, describing the true risk observed in all four cohorts (**Figure 4C**). The RAIS also can accurately separate the recurrence and recurrence-free CRC with tumor stage II/III after surgical resection. As illustrated in **Figure 4D**, patients in the high-risk group displayed a significantly higher fraction of recurrence (high-risk *vs.* low-risk: 22% *vs.* 6% in TCGA-CRC, 31% *vs.* 2% in GSE143985, 35% *vs.* 0% in GSE29621, and 21% *vs.* 0% in GSE92921; all *P <*0.05). The ROC analysis further suggested the RAIS possessed high accuracy for identifying CRC patients with recurrence in all four cohorts (**Figure 4E**). Taken together, the RAIS signature presented stable and excellent performance in evaluating RFS in patients with stage II/III CRC after surgical resection.

### Clinical and Molecular Characteristics of RAIS

In order to characterize the clinical significance of RAIS, we explored the relationship between clinical features and RAIS. As shown in **Figures S2**–**S5**, most clinical features, including age, sex, tumor stage, microsatellite instability, adjuvant chemotherapy, and *KRAS* mutation, were not significantly different between the high and low-risk groups in all four cohorts. In CRC, the *TP53* mutation is a key step driving the transition from adenoma to adenocarcinoma, and it is associated with adverse clinical outcomes (35). Obviously, in four cohorts, patients in the high-risk group had consistently higher proportion of *TP53* mutation compared with the low-risk group (all *P <*0.05) (**Figure S2**, **S3**, and **S5**), and the risk score was predominantly higher in patients with *TP53* mutation (all *P <*0.01) (**Figure S6**).

### TIME Landscape and Immune Checkpoints Profiles of RAIS

**Figure 5:**
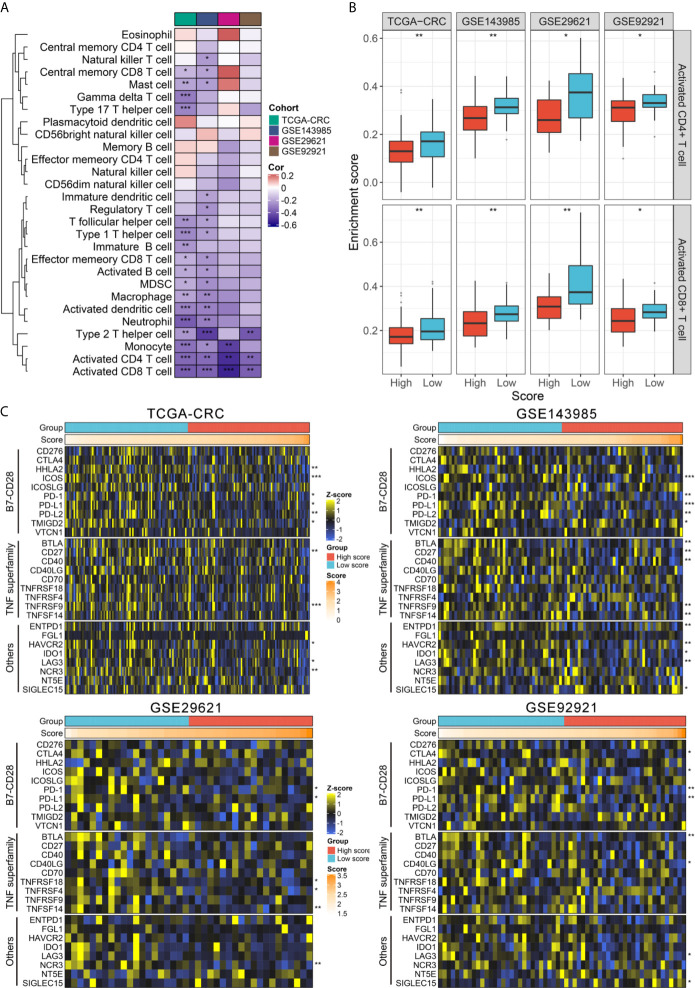
TIME landscape and immune checkpoints profiles of RAIS in four cohorts. **(A)** The correlation analysis between RAIS and 28 immune cells infiltration abundance. **(B)** The distribution difference of activated CD4+/CD8+ T cells infiltration between the high-risk and low-risk groups. **(C)** Four heatmaps of 27 immune checkpoints profiles in high-risk and low-risk groups. *P < 0.05, **P < 0.01, ***P < 0.001.

Since the establishment of RAIS was based on immune-related genes, we hypothesized that there were differences in the immune characterization between two recurrence-risk groups. First, the IPS score was utilized as a general index of immune activation in tumor tissues. Patients in the low-risk group displayed a higher IPS z-score compared with the high-risk group (all *P <*0.05) (**Figure S7A**). Next, ESTIMATE software was employed to infer the fraction of stromal and immune cells. In line with the IPS results, the low-risk group scored better in the immune category (all *P <*0.05) (**Figure S7B**). To gain more detailed insights into this issue, we applied the ssGSEA approach to quantify the infiltration abundance of different immune cell populations. Overall, the immune cells infiltration was more abundant in the low-risk group (**Figure S8A**), suggesting that their immune activity and immune response would be more active. Also, we could observe the risk score had broad negative relationships with different immune cell types (**Figure 5A**). Of note, there were significant and stable correlations between risk score and activated CD4+ T cell and CD8+ T cell in four cohorts (all *P <*0.01) (**Figure 5A**). Patients in the low-risk group possessed higher infiltration abundance of activated CD4+ T cell and CD8+ T cell (all *P <*0.05) (**Figure 5B**).

Furthermore, we extended our analysis to encompass 27 immune checkpoint members, including B7-CD28 family (*PD-L1*, *PD-L2*, *PD-1*, *CTLA4*, *CD276*, *HHLA2*, *ICOS*, *ICOSLG*, *TMIGD2*, and *VTCN1*) (36), the TNF superfamily (*BTLA*, *CD27*, *CD40*, *CD40LG*, *CD70*, *TNFRSF18*, *TNFRSF4*, *TNFRSF9*, and *TNFSF14*) (37), and several other molecules (*ENTPD1*, *FGL1*, *HAVCR2*, *IDO1*, *LAG3*, *NCR3*, *NT5E*, and *SIGLEC15*) (38, 39), and the results for four cohorts were presented in **Figure 5C**. In total, we observed that two molecules, including *PD-1* and *PD-L1*, were significantly upregulated in the low-risk group in four cohorts (all *P <*0.05) (**Figure S8B**). Consistent with this, the risk score was negatively correlated with the expression of *PD-1* and *PD-L1* (all *P <*0.05) (**Figure S8C**). Previous study has demonstrated patients with high expression of *PD-1* and *PD-L1* would benefit more from pembrolizumab and nivolumab (40, 41). Hence, to predict their putative response to ICB, two bioinformatics tools, TIS and TIDE, were used. As shown in **Figure S9**, patients in the low-risk group displayed the higher TIS score and the lower TIDE score (all *P <*0.05), suggesting that they were more likely to yield considerable clinical benefit from ICB therapy.

### Validation of RAIS in an Independent Cohort From Frozen Tissue Samples

**Figure 6:**
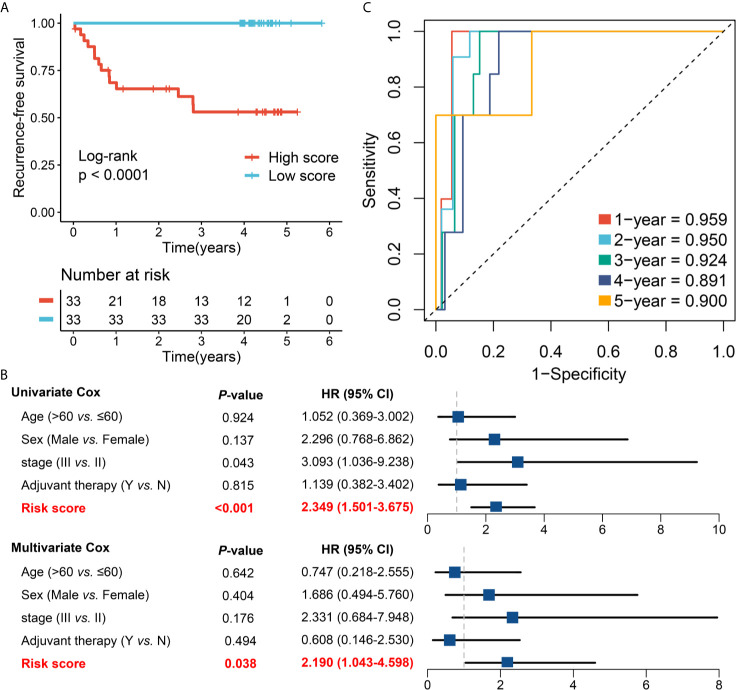
Validation of our discovery in a clinical in-house cohort. **(A)** Kaplan-Meier curves of RFS according to the RAIS. **(B)** Univariate and multivariate Cox regression analysis of the risk score. **(C)** Time-dependent ROC analysis for predicting RFS at 1~5 years.

To further verify the performance of our 12-gene RAIS model into a clinically translatable tool, we next evaluated the expression of these genes in a clinical cohort of 66 CRC patients by conducting qRT-PCR assay. The expression heatmap of the 12 selected IRGs and the distribution of risk scores were illustrated in **Figure S10A**. Consistently, the Kaplan-Meier survival analysis demonstrated that patients with high score displayed the dramatically poor RFS (HR =2.299, 95%CI =1.478~3.577, log-rank *P <*0.0001) (**Figures 6A, B**). After controlling for confounding variables (including age, sex, stage, and postoperative chemotherapy), the RAIS remained the statistical significance (HR = 2.190, 95%CI =1.043~4.598, *P <*0.05) (**Figure 6B**). ROC analysis showed the pinpoint accuracy of RAIS: the AUCs for predicting RFS at 1~5 years was 0.959, 0.950, 0.924, 0.891, and 0.900, respectively (**Figure 6C**). Similarly, the C-index reached 0.939 (95%CI =0.884~0.994). The calibration plot further displayed the predicted probabilities of RFS at 1~5 years accurately describing the true risk observed (**Figure S10B**). In addition, we also found that patients in the low-risk group might have more abundance of T cells (CD4+ and CD8+), and higher expression of *PD-1* and *PD-L1* (**Figure S10C**). Taken together, the results from a clinical in-house cohort supported that our discovery and in-silico validation cohort findings, which validated and confirmed that our RAIS model was quite robust, and can serve as an independent predictor of recurrence in stage II/III CRC.

## Discussion

CRC is a highly heterogeneous tumor that possesses complex biological processes, for which post-operative surveillance and therapeutic regimens are necessary to be tailored to generate an optimal outcome for each patient. Nevertheless, a considerable proportion of stage II/III CRC patients not only derive benefit from 5-FU-based adjuvant chemotherapy but also display drug reactions (5). A limitation of the current AJCC stage system is that patients in the same stage have distinct clinical outcomes, which leads to latent under- or over-treatment. Herein, developing a novel classifier that can be routinely implemented into clinical practice is critical for identifying those early-stage patients who are at high recurrence-risk and who might thus benefit from adjuvant chemotherapy. Considering various immune responses have close connections with the occurrence and progression of CRC (23, 25), we believe that introducing immune-based parameters in the clinical management of early-stage CRC is a high priority. Despite an international study recently proposed an index termed Immunoscore^®^ that presented stable and independent predictive of prognosis, it only considered two adaptive immune cells and showed a moderate accuracy. We hypothesized that a signature with high performance could be developed according to the global immune milieu. With the development of artificial intelligence and bioinformatics, an advanced machine learning algorithm can identify several key indicators that are most meaningful to predict clinical outcomes from a large number of genes (30), which is actually in line with the biological scale-free network which was dominated by a few hub nodes (42). Therefore, for the first time, we developed a novel signature (termed RAIS) to evaluate the recurrence-risk of patients with stage II/III CRC in multicenter cohorts based on the RNA expression of global immune genes. The reproducibility and powerful performance of RAIS in multiple independent cohorts and external qRT-PCR data not only prove that it is a robust and highly accurate model, but also is promising to be routinely implemented into clinical practice due to the advantages of high sensitivity and specificity, simplicity, and low cost of qRT-PCR.

In this study, we fitted a recurrence model consisting of 12 IRGs, including *MFI2*, *LTA*, *VEGFA*, *NPY*, *SHC3*, *RAG1*, *CASP1*, *NTF3*, *COCH*, *NMB*, *ERN1*, and *NLRP7*. All genes have been reported to be involved in the progression and TIME cross-talking of tumor. For example, *VEGFA* induces the expression of transcription factor *TOX* to drive T cell exhaustion (43), and the expression of *CASP1* is able to be repressed by *G9A* and further promotes tumor immune escape (44). Based on the 12 enrolled genes, we developed the RAIS model, which performed stably in predicting recurrent-risk of patients with stage II/III CRC. The prognostic meta-analysis showed that RAIS was an extremely vicious indicator of recurrence and was proven to be an independent factor after adjusting multiple clinical clinicopathologic features. More importantly, in four cohorts, RAIS demonstrated a high discrimination and calibration in predicting the recurrence-risk at 1~5 years. To prevent false positive results from sequencing data, we conducted another validation according to qRT-PCR results from 66 frozen CRC tissues with tumor stage II/III, confirming our prior findings and evaluating their practicality in different centers. As reported previously, patients with a high-risk score suggested dismal RFS, and thus might need to adjust therapy strategies or add additional adjuvant chemotherapy. For example, current guidelines recommend that a subset of stage II patients without “high-risk” traits do not require adjuvant chemotherapy (6), but when these patients show a high-risk score, using additional adjuvant chemotherapy might be essential.

Afterwards, we conducted a comprehensive analysis in the relationships between RAIS and clinical and molecular traits in four cohorts. RAIS was found to have no significant association with most of available traits. Of note, the risk score was dramatically higher in the *TP53* mutant CRC compared with wild type tumors. Based on the previous literature, patients with *TP53* mutation tended to display more aggressiveness and higher recurrence rate, which was in line with our finding (35). Strikingly, the interesting finding in this study was the relationship between RIAS and the prevalent immunotherapeutic biomarkers in stage II/III CRC. As is well-known, cancer immunotherapy represented by ICB has revolutionized the treatment of solid tumors, including a subset of CRC. Two monoclonal antibodies targeting *PD-1*, nivolumab and pembrolizumab, have demonstrated great efficacy in CRC with MSI-H mismatch repair deficiency (dMMR), and have been approved by FDA (40). In this study, patients in the low-risk group suggested an “immune-hot” subtype, displaying the rich infiltration of activated CD4+/ CD8+ T cells and the higher expression *PD-1*/*PD-L1*. High abundance of tumor-infiltrating lymphocytes (especially T cells) is not only a strong prognostic indicator, but also provides the backup resource for immunotherapy (45). Similarly, previous studies have reported that the high expression of *PD-1*/*PD-L1* correlates with favorable prognosis and better immunotherapeutic response in early-stage CRC (40, 41, 46). Hence, patients with the low-risk score suggested a potential benefit from immunotherapy. Two bioinformatic algorithms, TIS and TIDE, also showed the low-risk group might be more sensitive to immunotherapy relative to the high-risk group in four cohorts. Overall, patients with high-risk score might be not suitable for immunotherapy, due to potential ineffectiveness and immune-related adverse events (irAEs).

To the best of our knowledge, this is the first and most comprehensive study to date validating the prognostic accuracy of an immune signature in patients with stage II/III CRC undergoing surgical resection, based on the global immune genes. Prior to this study, a few reports established molecular signatures for predicting prognostic risk of CRC (47–50). In comparison with these studies, our work has several advantages and novelties: (1) The RAIS model was developed based on the recurrence rather than overall survival in patients with stage II/III CRC, which allowed it to accurately identify high-risk patients with early-stage CRC; (2) We performed comprehensive statistical approaches to evaluate the discrimination and calibration of the RAIS model, and our model remained stable and highly accurate performance at 1~5 years; (3) qRT-PCR was used to validate the performance of RAIS to ensure its robustness and clinical feasible; (4) We also demonstrated the TIME profiles and immune checkpoint landscape of RAIS, revealing its potential predictive value of immunotherapy. Despite the RAIS model is promising, some limitations should be acknowledged. First, all the samples from five centers were retrospective, and future validation of the RAIS model should be conducted in prospective fresh samples. Second, some clinical characteristics on public datasets were very inadequate, which thus had concealed the potential associations between RAIS and some clinical traits. Third, patients treated with immunotherapy were not examined in this study, so the performance of RAIS for predicting immunotherapeutic response was investigated indirectly. Further prospective study is still necessary.

In conclusion, we established a reproducible and powerful model for evaluating the recurrence-risk of patients with stage II/III CRC. Our study provides novel implications regarding immune profiles and stage II/III CRC recurrence. More importantly, the RAIS model may be a promising tool to optimize decision-making in adjuvant chemotherapy and immunotherapy, as well as tailor surveillance protocol for individual patients with stage II/III CRC.

## Data Availability Statement

The datasets presented in this study can be found in online repositories. The names of the repository/repositories and accession number(s) can be found in the article/**Supplementary Material**.

## Ethics Statement

The studies involving human participants were reviewed and approved by Ethics Committee of The First Affiliated Hospital of Zhengzhou University [December 19, 2019, TRN: 2019-KW-423]. The patients/participants provided their written informed consent to participate in this study.

## Author Contributions

ZL and XH designed this work. ZL, QD, ZS, JL, KX, and DJ integrated and analyzed the data. ZL, TL, LW, CG, and LL wrote this manuscript. ZL, JL, KX, ZS, and XH edited and revised the manuscript. All authors contributed to the article and approved the submitted version.

## Funding

This study was supported by the National Natural Science Foundation of China (81972663), Henan Province Young and Middle‐Aged Health Science and Technology Innovation Talent Project (YXKC2020037), and Henan Provincial Health Commission Joint Youth Project (SB201902014).

## Conflict of Interest

The authors declare that the research was conducted in the absence of any commercial or financial relationships that could be construed as a potential conflict of interest.

## Publisher’s Note

All claims expressed in this article are solely those of the authors and do not necessarily represent those of their affiliated organizations, or those of the publisher, the editors and the reviewers. Any product that may be evaluated in this article, or claim that may be made by its manufacturer, is not guaranteed or endorsed by the publisher.
