# Supplementary material for: Clinical Significance and Inflammatory Landscape of aNovel Recurrence-Associated Immune Signature in Stage II/III Colorectal Cancer
Source: Front Immunol. 2021 Jul 29;12:702594. doi: 10.3389/fimmu.2021.702594 (PMC8358813; doi:10.3389/fimmu.2021.702594)
Supplement: Supplementary file 1 [file DataSheet_1.docx]

**Supplementary material**

**Data processing**

**1. TCGA-CRC**

The colorectal cancer data were enrolled from The Cancer Genome Atlas (TCGA) cohorts TCGA-COAD (colon adenocarcinoma) and TCGA-READ (rectum adenocarcinoma). “Level 3” transcriptome data (RNA-Seq raw read count) and clinical information were retrieved from TCGA data portal (<https://portal.gdc.cancer.gov/>). Patients from TCGA were defined as TCGA-CRC cohort. The RNA-seq raw read count from TCGA database was converted to transcripts per kilobase million (TPM). A further log-2 transformation was performed due to RNA-seq data is often heavily right-skewed in the linear scale, which is more similar with the distribution of microarray data and more comparable between samples. The mRNAs with zero reads in all samples were further excluded.

**2. GSE143985**

Frozen tissue samples from 91 cases with stage II/III CRC who underwent curative surgery during the period of 2002–2003 at the Department of Surgery, National Defense Medical College Hospital were used for DNA microarray. No patient received preoperative chemotherapy or radiotherapy. RNA from 91 primary CRC samples were extracted and hybridized to Affymetrix human genome U133 plus 2.0 array. This study was conducted after obtaining approval from the internal review board of National Defense Medical College Hospital, Japan, and signed informed consent was obtained from all patients included in the study.

Data was normalized with robust multiarray averaging method in the affy package. After removing samples with no recurrence status or recurrence-free time, there were still 91 samples left in this dataset.

**3. GSE29621**

Tissues from patients undergoing surgical resection for colorectal cancer were collected with IRB approval and stored in the Tissue Procurement Core of American Tampa Moffitt Cancer Center. Histopathological examination of resected specimens (histological type, tumor size etc.) was performed by an experienced GI pathologist (D.C.). A representative section of tissue was cut and stained with hematoxylin and eosin to confirm the diagnosis. Microdissection of formalin fixed paraffin-embedded tissues was performed to isolate tumor sections (containing >90% tumor cells) for RNA extraction. Tumors that occurred in the background of genetic cancer syndromes or in the presence of inflammatory bowel disease were excluded. RNA from 65 primary CRC samples were extracted and hybridized to Affymetrix human genome U133 plus 2.0 array.

Data was normalized with robust multiarray averaging method in the affy package. After removing samples with no recurrence status or recurrence-free time, there were still 40 samples left in this dataset.

**4. GSE92921**

A total of 59 patients treated at the National Defense Medical College with resected stage II/III CC who had not received postoperative adjuvant chemotherapy were enrolled. RNA from 59 primary CRC samples were extracted and hybridized to Affymetrix human genome U133 plus 2.0 array. The protocols and procedures for this study were approved by the National Defense Medical College Center.

Data was normalized with robust multiarray averaging method in the affy package. After removing samples with no recurrence status or recurrence-free time, there were still 59 samples left in this dataset.

**5. qRT-PCR assay of 66 FFPE samples**

From January 2015 to December 2016, we collected a total of 66 frozen surgically resected CRC tissues with AJCC stage II/III at The First Affiliated Hospital of Zhengzhou University. Clinical staging of the specimens was based on NCCN (2019) guidelines. Detailed baseline data of CRC patients are displayed in Table S1. Total RNA was isolated from CRC tissues using RNAiso Plus reagent (Takara, Dalian, China) according to the manufacturer’s instructions. RNA quality was evaluated using a NanoDrop One C (Waltham, MA, USA), and RNA integrity was assessed using agarose gel electrophoresis. An aliquot of 1 µg of total RNA was reverse-transcribed into complementary DNA (cDNA) according to the manufacturer’s protocol using the miRNA reverse transcription Kit (TaKaRa BIO, Japan). All cDNA samples were prepared for qRT-PCR. This project was approved by the Ethics Committee Board of The First Affiliated Hospital of Zhengzhou University.

In the qRT-PCR analysis, the enrolled 12 genes in the RAIS signature and feature gene (including PD-L1, PD-1, CD4 and CD8A) were detected. qRT-PCR was performed using SYBR Assay I Low ROX (Eurogentec, USA) and SYBR® Green PCR Master Mix (Yeason, Shanghai, China). The expression value of the target genes was normalized to GAPDH, and then log2 transformed for subsequent analysis. The primer sequences of the included 12 genes and GAPDH were shown in Table S2.
