# Supplementary material for: Clinical Significance and Inflammatory Landscape of aNovel Recurrence-Associated Immune Signature in Stage II/III Colorectal Cancer
Source: Front Immunol. 2021 Jul 29;12:702594. doi: 10.3389/fimmu.2021.702594 (PMC8358813; doi:10.3389/fimmu.2021.702594)
Supplement: Supplementary file 2 [file DataSheet_2.docx]

Supplementary Figure

- Figure S1
- Figure S2
- Figure S3
- Figure S4
- Figure S5
- Figure S6
- Figure S7
- Figure S8
- Figure S9
- Figure S10


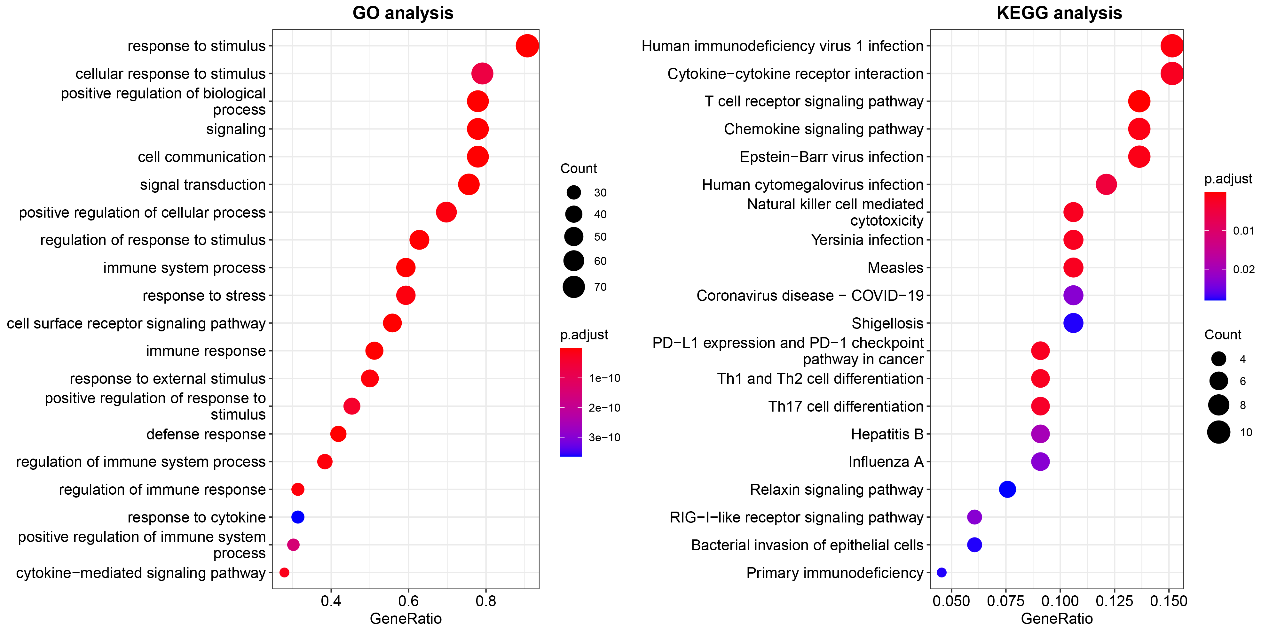


**Figure S1**. GO and KEGG enrichment analysis of 88 IRGs that stably associated with RFS.


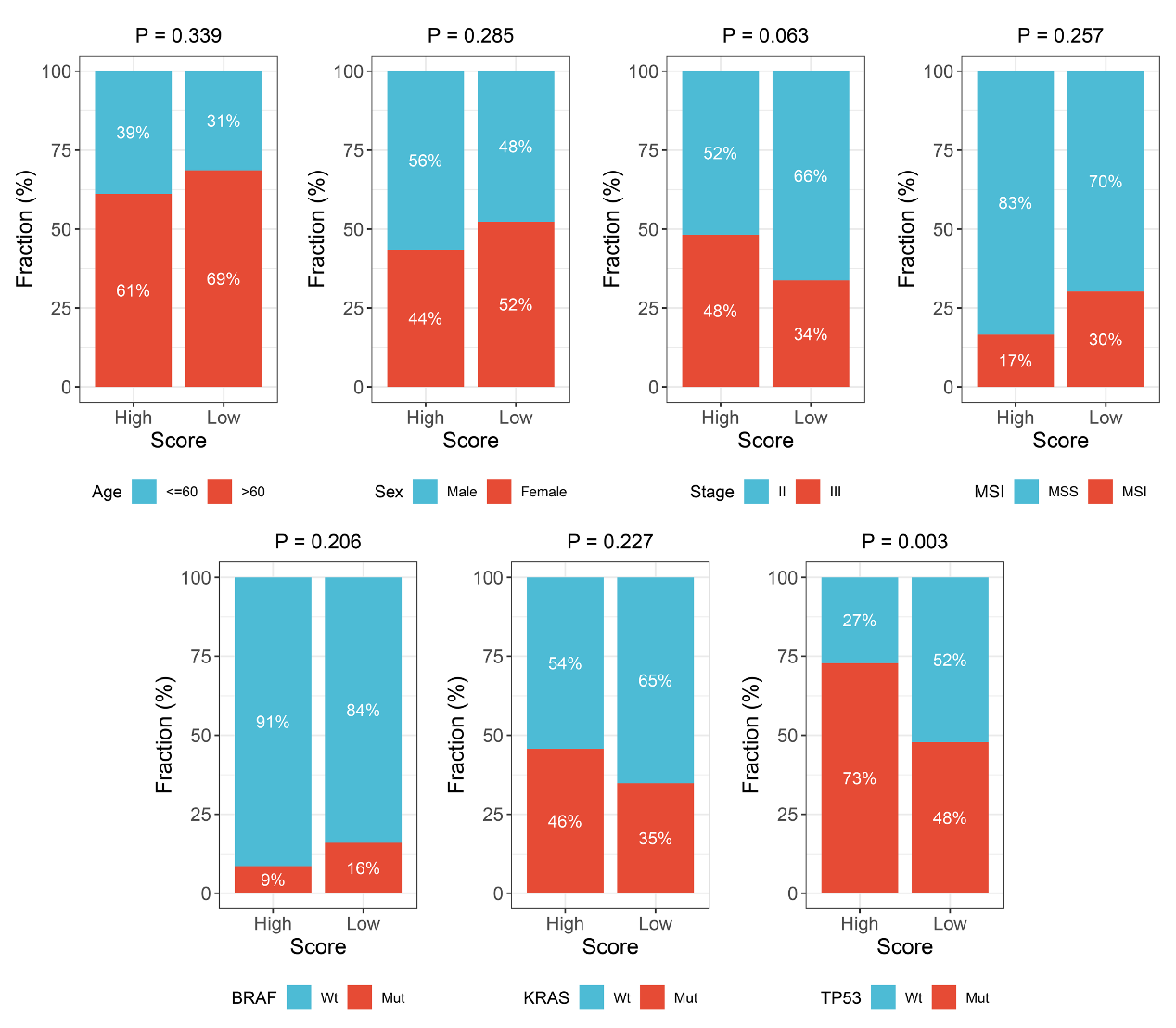


**Figure S2**. Clinical and molecular characteristics of RAIS in TCGA-CRC.


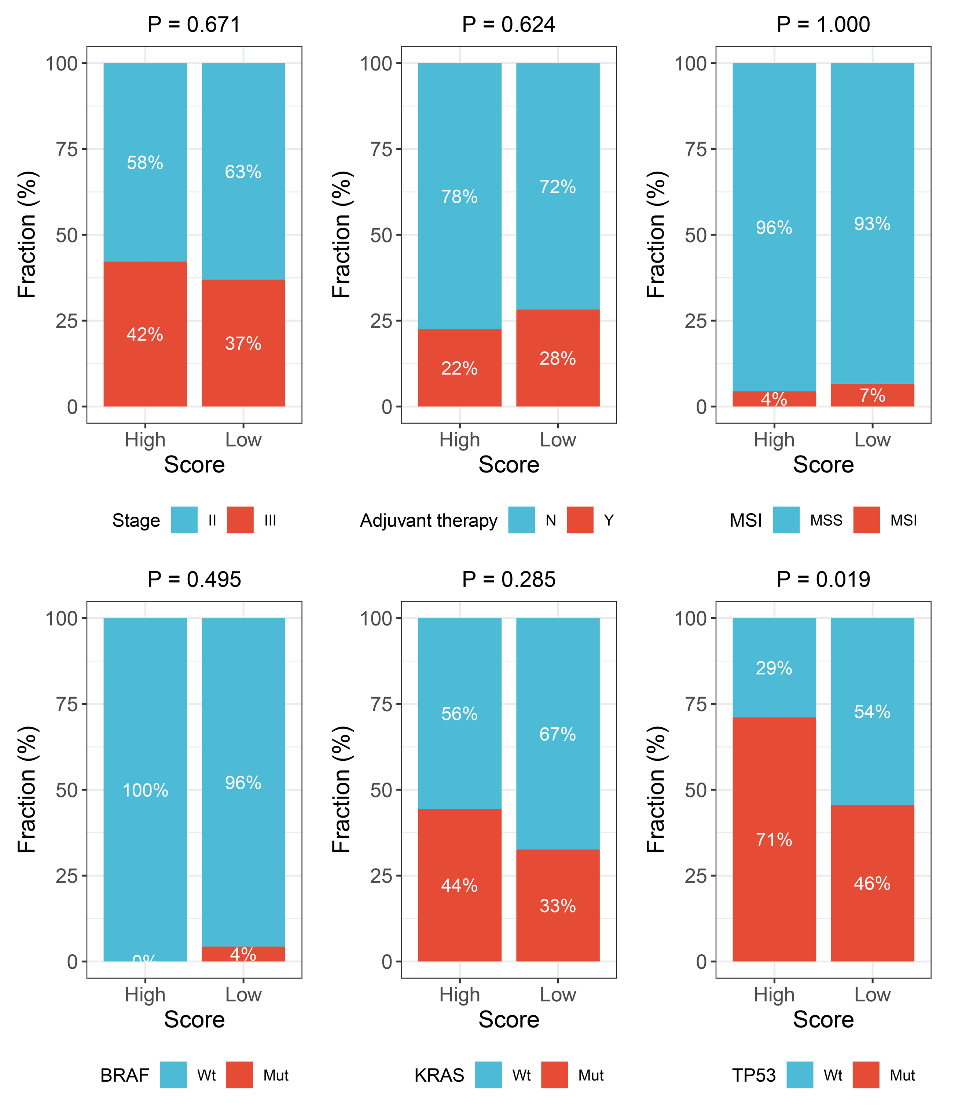


**Figure S3**. Clinical and molecular characteristics of RAIS in GSE143985.


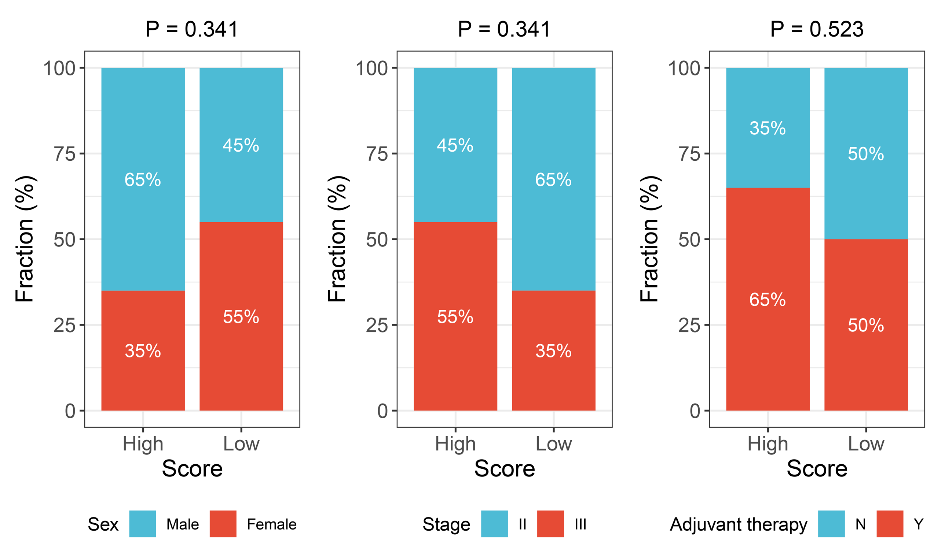


**Figure S4**. Clinical and molecular characteristics of RAIS in GSE29621.


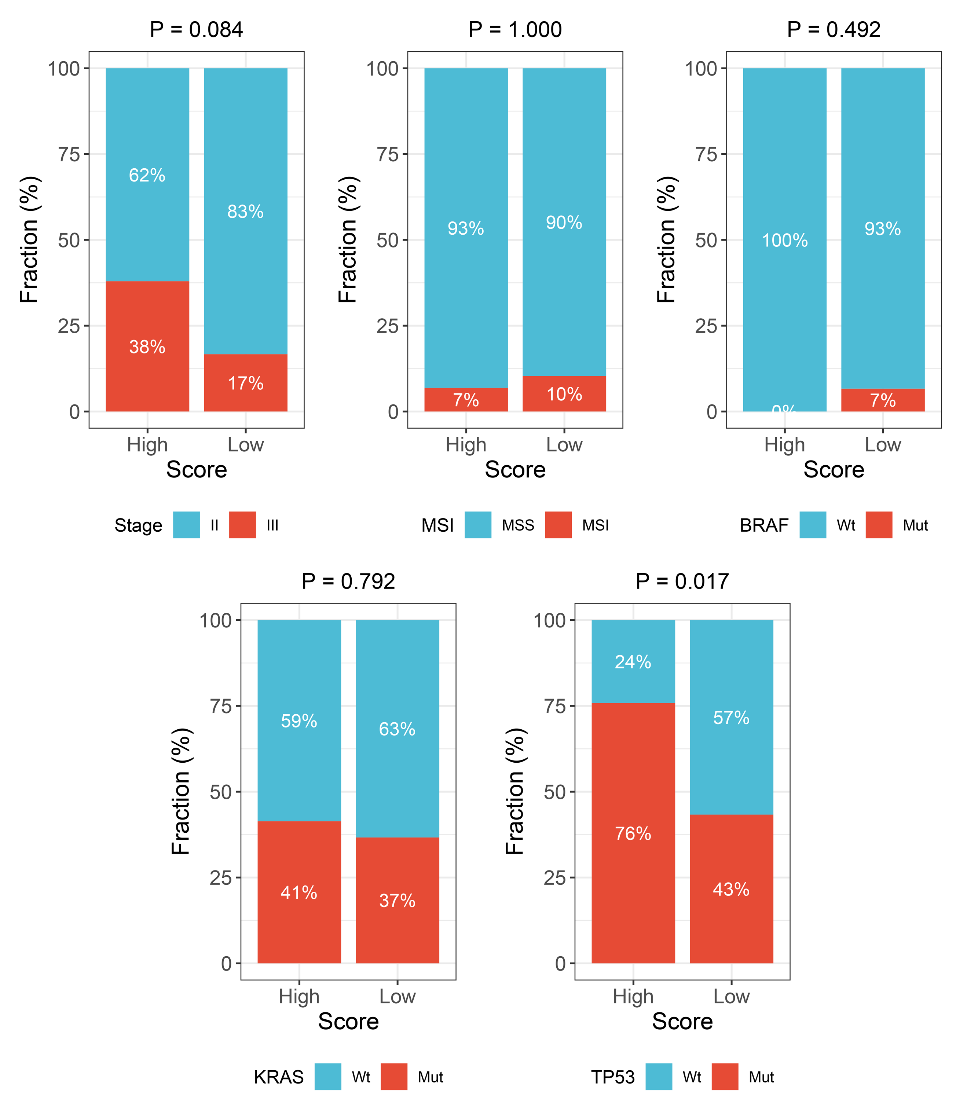


**Figure S5**. Clinical and molecular characteristics of RAIS in GSE92921.


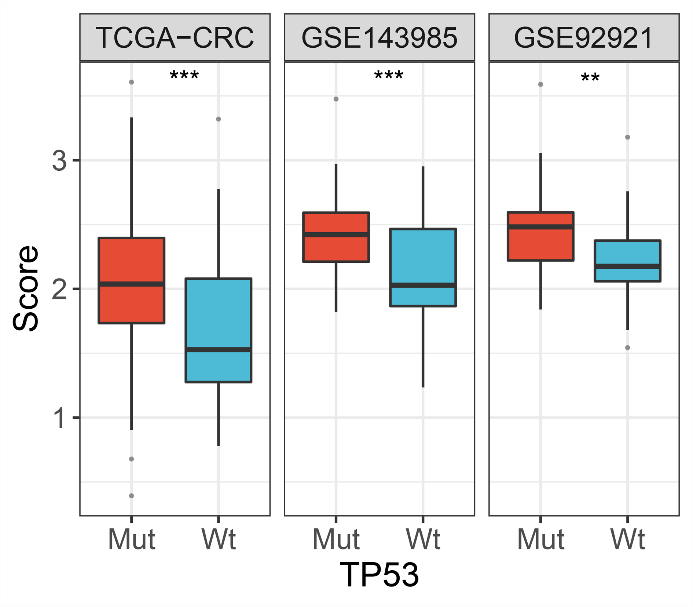


**Figure S6**. The distribution difference of risk score between TP53 mutant CRC and wild type tumors. **P <0.01, ***P <0.001.


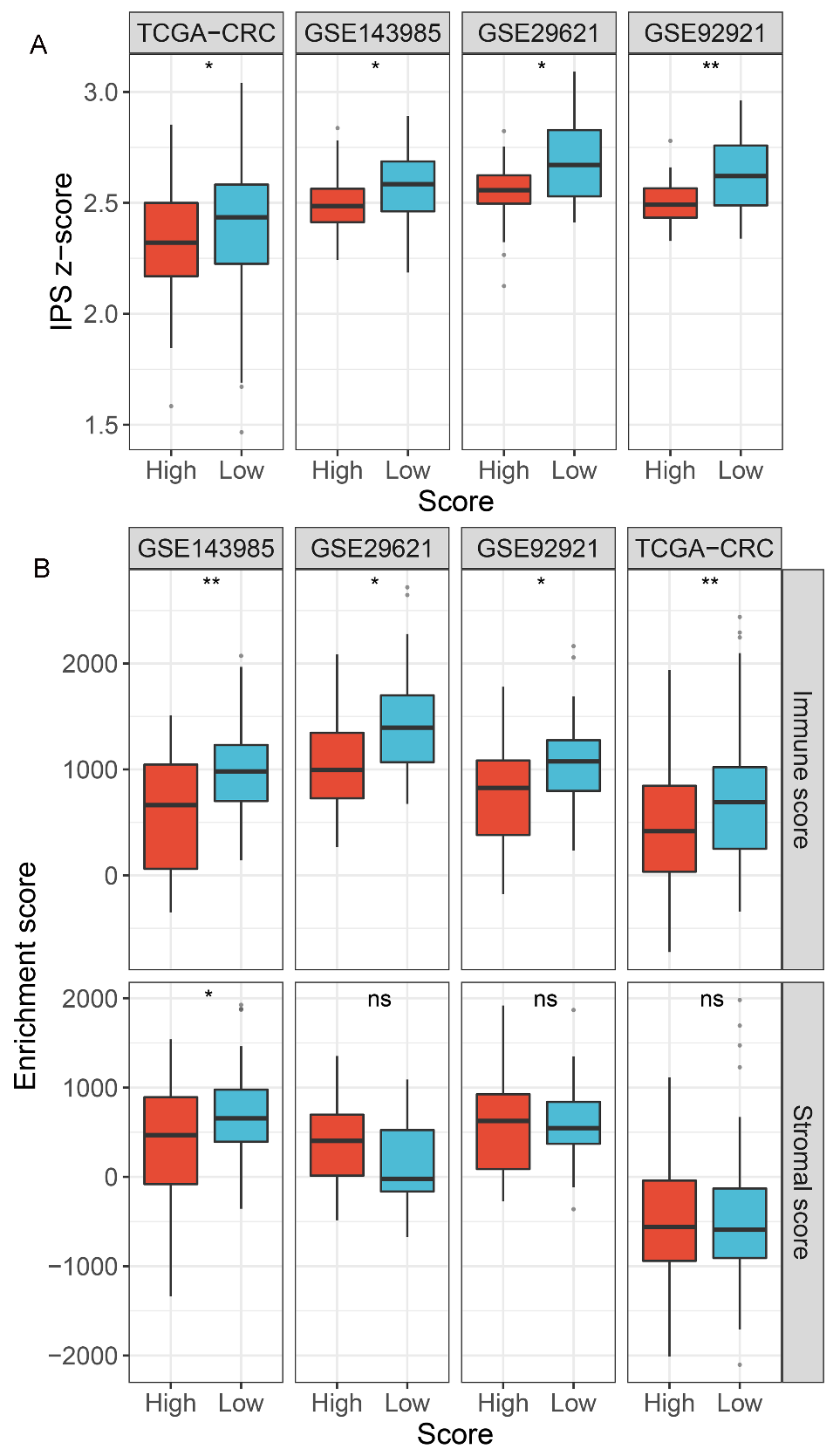


**Figure S7**. The IPS and ESTIMATE analysis of RAIS in four cohorts. A. The distribution difference of IPS z-score between the high-risk and low-risk groups. B. The distribution difference of immune and stromal enrichment score between the high-risk and low-risk groups. ^ns^P >0.05, *P <0.05, **P <0.01.


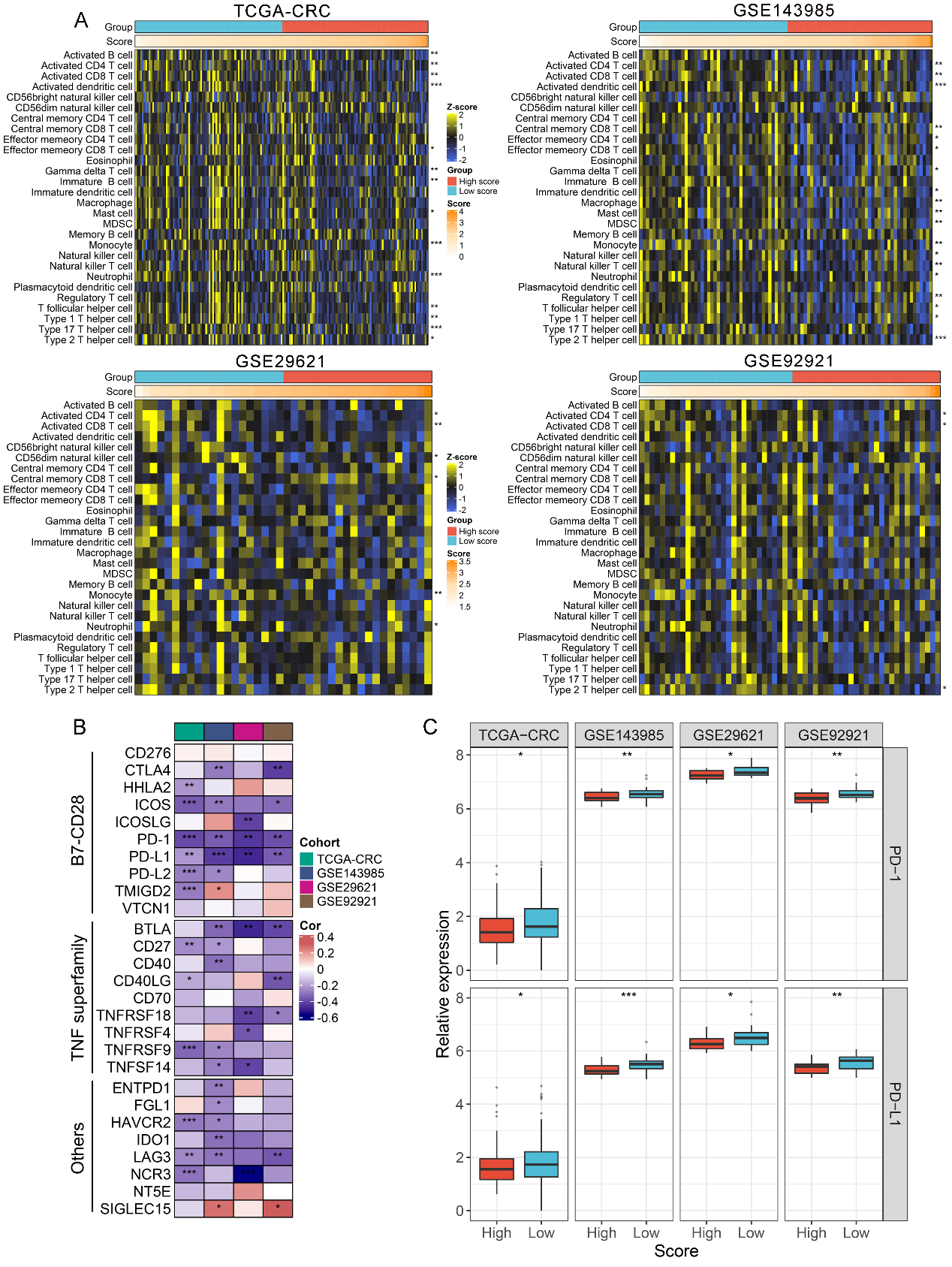


**Figure S8**. Immune cells and immune checkpoints analysis of RAIS in four cohorts. **A**. Four heatmaps of 28 immune cells infiltration in high-risk and low-risk groups. **B**. The correlation analysis between RAIS and 27 immune checkpoints profiles. **C**. The distribution difference of *PD-1*/*PD-L1* between the high-risk and low-risk groups. *P <0.05, **P <0.01, ***P <0.001.


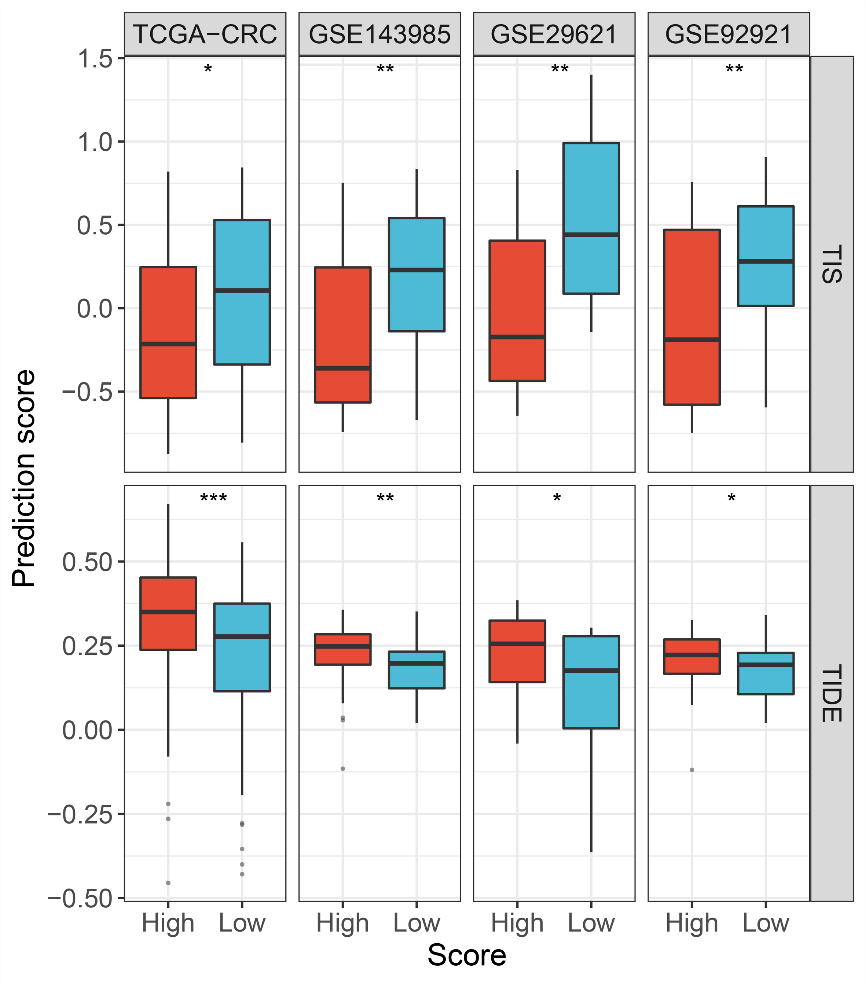


**Figure S9**. The distribution difference of TIS and TIDE prediction score between the high-risk and low-risk groups. *P <0.05, **P <0.01, ***P <0.001.


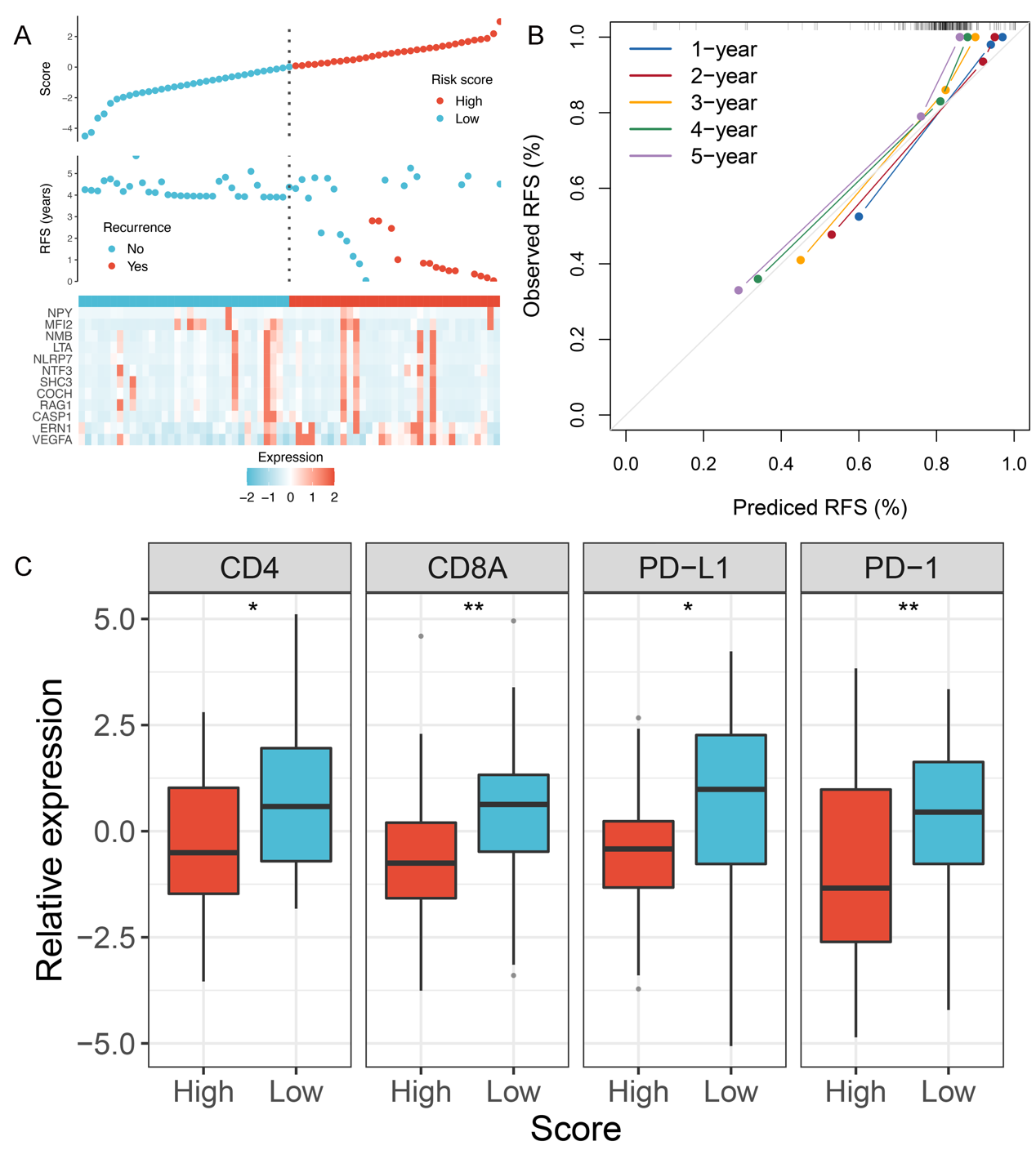


**Figure S10.** Validation of our discovery in a clinical in-house cohort. **A**. The distribution of risk score, recurrence status, and gene expression panel in our cohort. **B**. Calibration plots for comparing the actual probabilities and the predicted probabilities of RFS at 1~5 years. **C**. The distribution difference of *CD4*, *CD8A*, *PD-L1* and *PD-1* between the high-risk and low-risk groups. *P <0.05, **P <0.01.
