# Supplementary material for: Clinical Significance and Inflammatory Landscape of aNovel Recurrence-Associated Immune Signature in Stage II/III Colorectal Cancer
Source: Front Immunol. 2021 Jul 29;12:702594. doi: 10.3389/fimmu.2021.702594 (PMC8358813; doi:10.3389/fimmu.2021.702594)
Supplement: Supplementary file 3 [file Table_1.docx]

**Table S1**. Details of baseline information in TCGA-CRC, GSE143985, GSE29621, GSE92921, and qRT-PCR data from 66 samples, respectively.

| **Characteristics** | **TCGA-CRC** | **GSE143985** | **GSE29621** | **GSE92921** | **qRT-PCR assay** |
| --- | --- | --- | --- | --- | --- |
| **Recurrence** |  |  |  |  |  |
| No | 147 (86.0) | 76 (83.5) | 33 (82.5) | 53 (89.8) | 52 (78.8) |
| Yes | 24 (14.0) | 15 (16.5) | 7 (17.5) | 6 (10.2) | 14 (21.2) |
| **Stage** |  |  |  |  |  |
| II | 101 (59.1) | 55 (60.4) | 22 (55.0) | 43 (72.9) | 40 (60.6) |
| III | 70 (40.9) | 36 (39.6) | 18 (45.0) | 16 (27.1) | 26 (39.4) |
| **Age** |  |  |  |  |  |
| <60 | 60 (35.1) | —— | —— | —— | 32 (48.5) |
| ≥60 | 111 (64.9) | —— | —— | —— | 34 (51.5) |
| **Gender** |  |  |  |  |  |
| Male | 89 (52.0) | —— | 22 (55.0) | —— | 36 (54.5) |
| Female | 82 (48.0) | —— | 18 (45.0) | —— | 30 (45.5) |
| **Adjuvant chemotherapy** |  |  |  |  |  |
| No | —— | 64 (70.3) | 17 (42.5) | —— | 35 (53.0) |
| Yes | —— | 22 (24.2) | 23 (57.5) | —— | 31 (47.0) |
| Unavailable | —— | 5 (5.5) | 0 (0) | —— | 0 (0) |
| **Microsatellite status** |  |  |  |  |  |
| Stable (MSS) | 50 (29.2) | 85 (93.4) | —— | —— | —— |
| Instable (MSI) | 17 (10.0) | 5 (5.5) | —— | —— | —— |
| Unavailable | 104 (60.8) | 1 (1.1) | —— | —— | —— |
| **TP53** |  |  |  |  |  |
| Wt | 55 (32.2) | 38 (41.8) | —— | 24 (40.7) | —— |
| Mut | 84 (49.1) | 53 (58.2) | —— | 35 (59.3) | —— |
| Unavailable | 32 (18.7) | 0 (0) | —— | 0 (0) | —— |
| **KRAS** |  |  |  |  |  |
| Wt | 83 (48.5) | 56 (61.5) | —— | 36 (61.0) | —— |
| Mut | 56 (32.7) | 35 (38.5) | —— | 23 (39.0) | —— |
| Unavailable | 32 (18.7) | 0 (0) | —— | 0 (0) | —— |
| **BRAF** |  |  |  |  |  |
| Wt | 122 (71.3) | 89 (97.8) | —— | 57 (96.6) | —— |
| Mut | 17 (9.9) | 2 (2.2) | —— | 2 (3.4) | —— |
| Unavailable | 32 (18.7) | 0 (0) | —— | 0 (0) | —— |
